# Effects of obesity on the healing of bone fracture in mice

**DOI:** 10.1186/s13018-018-0837-7

**Published:** 2018-06-08

**Authors:** Feng Gao, Tian-Run Lv, Jin-Chun Zhou, Xiao-Dong Qin

**Affiliations:** Department of Orthopedic Surgery, First Affiliated Hospital, Nanjing Medical University, No. 300 Guangzhou Road, Gulou District, Nanjing, 210029 Jiangsu China

**Keywords:** Obesity, Bone healing, CGRP, FGF, TGF-β1, TNF-α

## Abstract

**Background:**

Obesity affects bone health to varying degrees, depending on the skeletal site (weight-bearing or non-weight-bearing) and compartment (cortical or trabecular), and is a risk factor for orthopedic disorders, including bone fractures. However, the effect and mechanisms of obesity on healing of bone fracture is little understood.

**Methods:**

The healing bone fractures of the tibia in genetically obese mice was evaluated relative to normal mice at weekly intervals for 28 days using X-ray scans, hematoxylin and eosin (H&E) stain, and alcian blue (AB) stain. Plasma concentrations of relevant proteins were also compared via enzyme-linked immunosorbent assay (ELISA). These included calcitonin gene-related peptide (CGRP), fibroblast growth factor (FGF), transforming growth factor beta 1 (TGF-β1), and tumor necrosis factor-α (TNF-α).

**Results:**

Bone fracture healing was delayed in the obese mice compared with the control group of normal mice, based on X-ray, H&E stain, and AB stain analysis. This was accompanied with significantly low plasma CGRP, FGF, and TGF-β1 (ELISA). However, TNF-α was significantly higher in obese mice compared with the control.

**Conclusion:**

Bone fracture healing was significantly slower in the obese mice, relative to that of normal mice. The lower levels of CGRP, FGF, and TGF-β, and higher level of TNF-α, observed in obese mice may contribute to this observed delay in fracture healing.

## Background

Obesity is a complex disorder in which excess body fat has accumulated to a body mass index ≥ 30 kg/m^2^ [1], due to an energy imbalance between calories consumed and calories burned. According to the World Health Organization, in the year 2014 worldwide more than 600 million people were obese [2], with the prevalence doubling from 1980 to 2014 [2]. In developed countries, the prevalence of obesity is higher—for example, in the USA in 2007, 33% of men and 35% of women were obese.

Obesity is a significant contributor to many chronic disorders such as hypertension, dyslipidemia, type 2 diabetes mellitus, coronary heart disease, and certain cancers [3]. However, it has been considered that obesity may be beneficial to bone health, because of the well-established positive effect of mechanical loading (here, body weight) on bone formation. Controversially, fat accumulation due to obesity is detrimental to bone mass. Research has also indicated that obesity may affect bone metabolism by any of the following effects: increasing adipocyte differentiation and fat accumulation; decreasing osteoblast differentiation and bone formation; increasing circulating and tissue proinflammatory cytokines (promoting osteoclast activity and bone resorption); upregulating proinflammatory cytokine production; and interfering with intestinal calcium absorption, thereby decreasing calcium availability for bone formation [1, 3–12]. Less understood are the effects and mechanisms of obesity on bone fracture healing.

The present study investigated the effects of obesity (alone) on the healing of bone fracture, using a murine model of specific-pathogen-free (SPF) B6.Cg-*Lep*^ob^/J ob/ob male mice (obese). We also included SPF normal-weight healthy male mice as the control group for comparison. X-ray scans, hematoxylin and eosin (H&E) stain, and alcian blue (AB) stain were used to study the progress of bone healing at the following post-fractural timepoints: days 0, 7, 14, 21, and day 28. The plasma concentrations of relevant proteins were also evaluated at the same timepoints. The relevant proteins included calcitonin gene-related peptide (CGRP), fibroblast growth factor (FGF), transforming growth factor beta 1 (TGF-β1), and tumor necrosis factor-α (TNF-α).

Our results suggest that bone fracture healing was significantly slower in the obese mice, relative to that of normal mice. The lower levels of CGRP, FGF, and TGF-β, and higher level of TNF-α, observed in obese mice may contribute to this observed delay in fracture healing.

## Methods

### Materials

Pentobarbital sodium was purchased from Sigma-Aldrich (St. Louis, USA). Saline (0.9%) was obtained from Shandong Kangning Pharmaceutical Industry (Liaocheng, China). Ethylenediaminetetraacetic acid (EDTA), wax, and liquid paraffin were ordered from Shanghai Aladdin Bio-Chem Technology (Shanghai, China). Chloral hydrate and formalin were purchased from Sinoreagent (Shanghai, China), and neutral balsam from Shanghai Huashen Recover Equipment (Shanghai, China). Ethanol was acquired from Wuxi Yasheng Chemical (Jiangsu, China); xylene, hydrogen peroxide, and methanol from Wuxi Zhanwang Chemical (Jiangsu, China). Bovine serum albumin was purchased from Biosharp (Hefei, China). The 3,3′-diaminobenzidine (DAB) staining kit was from Boster Biotechnology (Wuhan, China). The H&E staining kit was purchased from Beyotime Biotechnology (Shanghai, China). Enzyme-linked immunosorbent assay (ELISA) kits for CGRP, TGF-β1, FGF, and TNF-α were purchased from Feiya Biotechnology (Jiangsu, China). AB stain was purchased from Solarbio (Beijing, China).

### Imposing tibia fractures

The Institutional Animal Care and Use Committee approved the use of mice in this study, which also complied with the guidance for animal use set forth by the National Institutes of Health. Twenty SPF male C57BL/6J mice (normal healthy control group; 6–8 weeks old) and 20 SPF male B6.Cg-*Lep*^ob^/J ob/ob mice (obese group; 6–8 weeks old) were purchased from the Jackson Laboratory (Bar Harbor, ME, USA). These mice were checked after arrival to ensure that they were not infected with any diseases. They were housed at 18–26 °C and 40–70% humidity with freely accessible water and food in the animal center. The body weights of the mice in both groups were monitored throughout this study. Both groups gained weight during the study, but the obese mice gained much more weight than the control mice (Table 1).

**Table 1:** Body weights of normal and obese mice at various days post-surgery (g)

	0	7	14	21	28
C57BL/6J	21.8 ± 0.9	20.1 ± 0.5	21.6 ± 0.6	22.9 ± 0.6	24.3 ± 0.8
ob/ob	34.1 ± 1.2	32.0 ± 1.5	33.4 ± 1.3	35.4 ± 1.3	36.8 ± 0.9

To implement fractures of the tibia, each mouse was completely sedated with 1% pentobarbital sodium and placed supine on a surgical table (Taizhou Xintai Medical Equipment Manufacturing, Jiangsu, China). The hair on the right caudal limb was shaved, and the limb was disinfected. A longitudinal incision (0.5 cm) was made below the right keen joint. The muscle and fascia were separated from the tibia, and the tibial shaft was cut at the caudate one-third using a bone saw. The tibial bone surfaces were immediately irrigated with sterilized 0.9% saline. A stainless steel intramedullary rod 1.0 (Jiangzhou Medical Devices, Jiangsu, China) was inserted to reconnect the broken tibial bones. The incisions were sutured via layer by layer.

All the mice had free activities and free access to food. A blood sample was collected from each mouse before the surgery (baseline). After the surgery, the mice in each group (obese and normal control) were randomly assigned to four sub-groups (*n* = 5 for each subgroup) to be examined at 7, 14, 21, and 28 days, respectively.

### X-ray

X-ray radiographic analysis has been widely used to ensure the fracture pattern and the position of the fixation needle, and qualitatively examine fracture healing [13]. At the pre-set post-operative timepoints (days 7, 14, 21, and 28), one subgroup (5 mice) from each group of mice (i.e., obese and control) were selected and filmed using LX-24HA X-ray (Konica Minolta, Japan) at 30 kV and 8 mA before they were sampled for blood and then killed.

### ELISA immunohistochemistry

At each postoperative timepoint (days 0, 7, 14, 21, and 28), blood samples were withdrawn from the designated subgroup (5 mice) selected for X-ray imaging from the obese and control mice and analyzed using ELISA [14, 15]. Briefly, the selected mice were anesthetized via intraperitoneal injection of 10% chloral hydrate. After an eyeball was removed, 0.2–0.6 mL of blood was collected from the eye socket of each mouse using a 1.5 mL centrifuge tubes (EP). Each whole blood sample was centrifuged at 4000 rpm for 10 min to obtain the blood plasma sample. The resulting plasma samples were stored at − 80 °C before ELISA. The plasma concentrations of CGRP, TGF-β1, FGF, and TNF-α were quantified using the corresponding ELISA kits, in accordance with the manufacturers’ instructions.

### H&E staining

At each postoperative timepoint (days 0, 7, 14, 21, and 28), after the X-ray imaging and blood collection (described above), the selected mice were killed. Some fractured tibial bone was harvested from each mouse for H&E staining. Briefly, two specimens (0.5 cm long, each) were cut from each freshly collected bone sample, one from each side of the fractured bone and starting from the fracture site. The fresh specimens were fixed in 10% neutral formalin solution for 48 h. The fixed specimens were soaked in 18% EDTA solution, decalcified using a microwave until the specimens were soft enough for a needle to penetrate, then washed under running water for 12 h.

The resulting specimens were dehydrated, cleared, dipped in wax, and then processed with an embedding machine. The specimens were embedded using liquid paraffin in the presence of a base film and plastic-covered box. The embedded specimens were sliced using a microtome, to 5-μm slices. The slices were de-waxed, rehydrated, and stained using an H&E staining kit in accordance with the manufacturer’s instructions. The stained slices were dehydrated, sealed using neutral balsam, and examined under an Olympus IX71 microscope (Tokyo, Japan).

### AB staining

The protocol for specimen collection and preparation and staining with AB, and viewing slides, was identical to the protocol for H&E staining, except that AB was used for staining.

### Statistical analysis

Each experiment was repeated ≥ 3 times. All numerical experimental data are presented as mean ± standard deviation. Statistical analyses were performed using one-way analysis of variance and the *t* test, with SPSS 19.0 software. Statistically significant differences between the obese and control groups were considered whenever the *P* value was < 0.05.

## Results

### Delayed bone fracture healing for mice

**Fig. 1:**
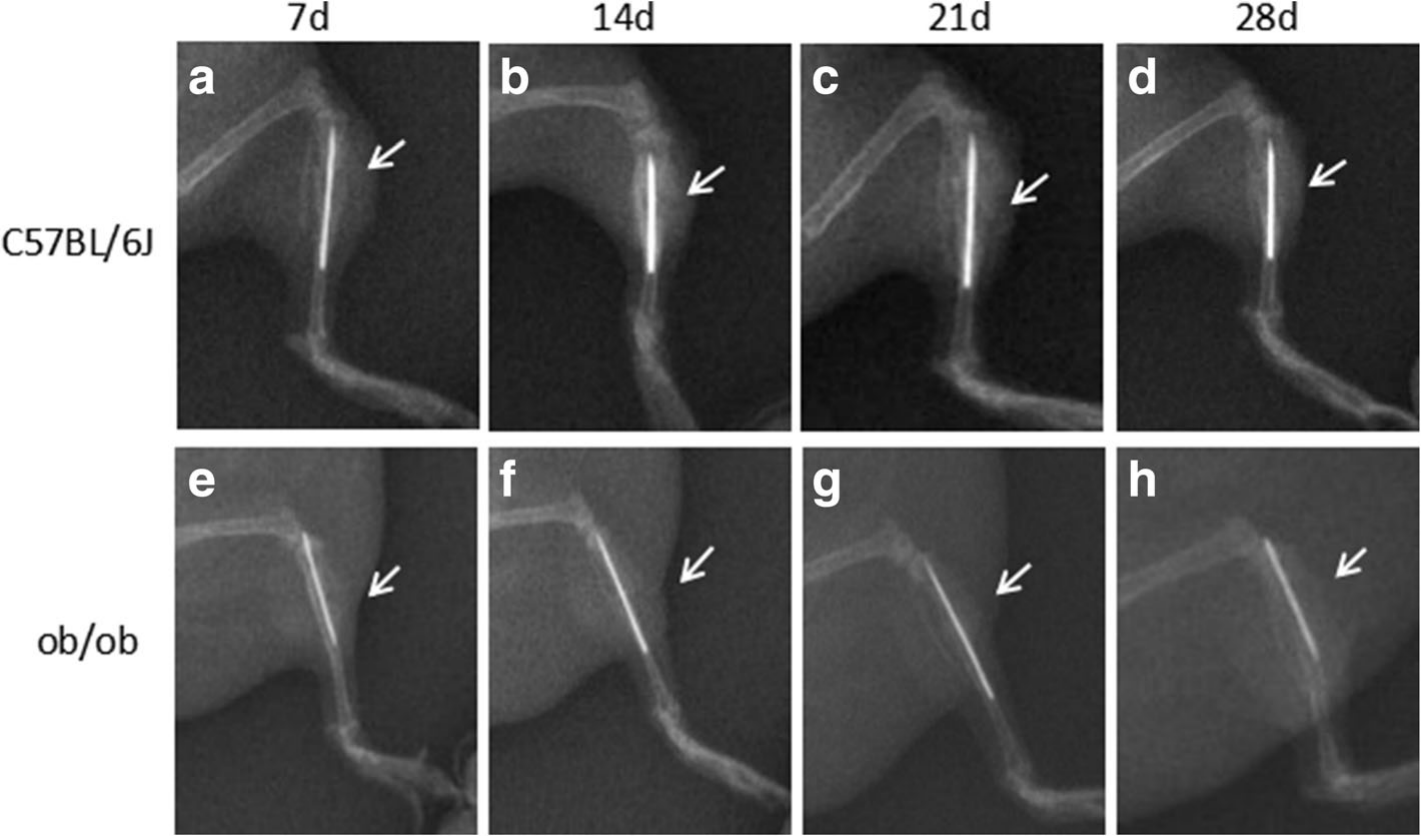
Representative X-ray images of bone fracture sites for normal control mice (**a**, **b**, **c**, and **d**) and obese mice (**e**, **f**, **g**, and **h**) at day 7 (**a** and **e**), day 14. (**b** and **f**), day 21 (**c** and **g**), and day 28 (**d** and **h**) of post-operation. Arrows point to the fracture sites

X-ray images were obtained of the fracture sites of the obese and normal control mice at post-fracture days 7, 14, 21, and 28 (Fig. 1). No dislocation was observed of the intramedullary rods in either group at any timepoint. Both groups of mice had clear fracture lines at the fracture site at postoperative day 7 (Fig. 1a, e).

At postoperative day 14, the control group showed a large number of blurred shadows, which indicated callus formation (Fig. 1b). The obese mice showed no obvious blurred shadows around the fracture sites, suggesting little or no formation of callus (Fig. 1f). At postoperative day 21, the control group showed a large number of continuous blurred images (Fig. 1c), and on day 28, continuous cortical bone at the fracture sites (Fig. 1d). In the obese group, on the corresponding postoperative days (21 and 28) there was less indication of callus and there was no continuous cortical bone at the fracture sites (Fig. 1g, h).

### Cell activities at bone fracture sites

**Fig. 2:**
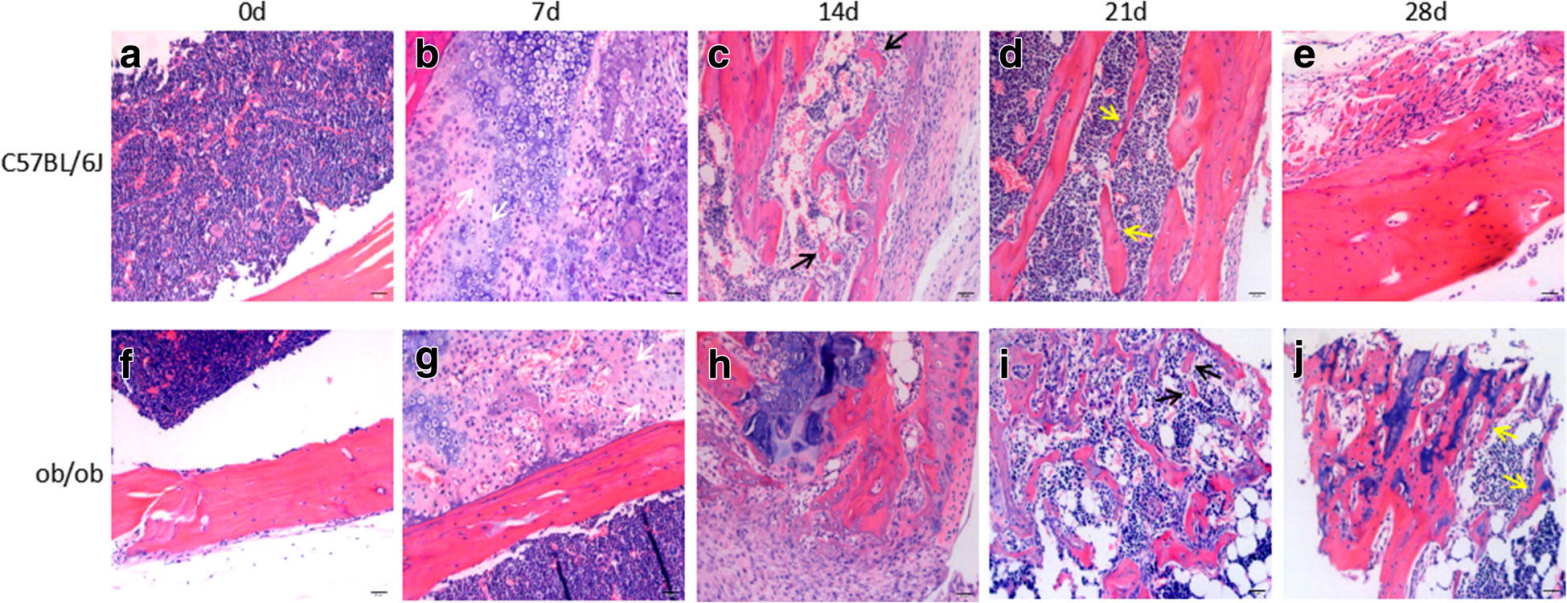
Representative optical images of H&E-stained bone slides from fracture sites of normal mice (**a**, **b**, **c**, **d**, and **e**) and obese mice (**f**, **g**, **h**, **i**, and **j**) at day 0 (**a** and **f**), day 7 (**b** and **g**), day 14 (**c** and **h**), day 21 (**d** and **i**), and day 28 (**e** and **j**). Magnification × 500 and scale bar 20 μm. White arrows point to undifferentiated mesenchymal cells. Black arrows point to new trabecular bone. Yellow arrows point to new callus

Optical images of H&E stained bone specimens were obtained at each post-fracture timepoint for each group of mice (Fig. 2). At postoperative day 7, the H&E stained bone specimens of all the mice in each group had a large number of undifferentiated mesenchymal cells at the fracture sites (Fig. 2b, g). In addition, the control mice had a large number of chondrocytes that were in the resting period or proliferative phase at the fracture sites (Fig. 2b), while the obese mice had relatively few chondrocytes at the fracture sites (Fig. 2g).

At postoperative day 14, the normal mice had a large number of chondrocytes and much collagen tissue at the fracture sites, and a small amount of new cancellous bone trabeculae was also observed (Fig. 2c). At the same timepoint, the obese mice had a large number of chondrocytes (mainly hypertrophic chondrocytes) and much collagen tissue at the fracture sites, and a small amount of new cancellous bone trabeculae (Fig. 2h).

At postoperative day 21, in the control mice, only a few chondrocytes and no collagen tissue were observed at the fracture sites, but trabecular bones were visible (Fig. 2d). In the obese mice, a few chondrocytes and collagen tissue were observed at the bone fracture sites, and trabecular bones could be seen (Fig. 2i).

At postoperative day 28, in the normal mice, the fracture ends were connected by bone scabs, which were filled with well-arranged trabecular bone and osteoblasts (Fig. 2e). In the obese mice, a large number of osteoblasts and bone cells in the bone mass were observed at the bone fracture ends, with a reduction in cartilage callus and increase in bone callus as compared with the obese mice on day 21 (Fig. 2j).

**Fig. 3:**
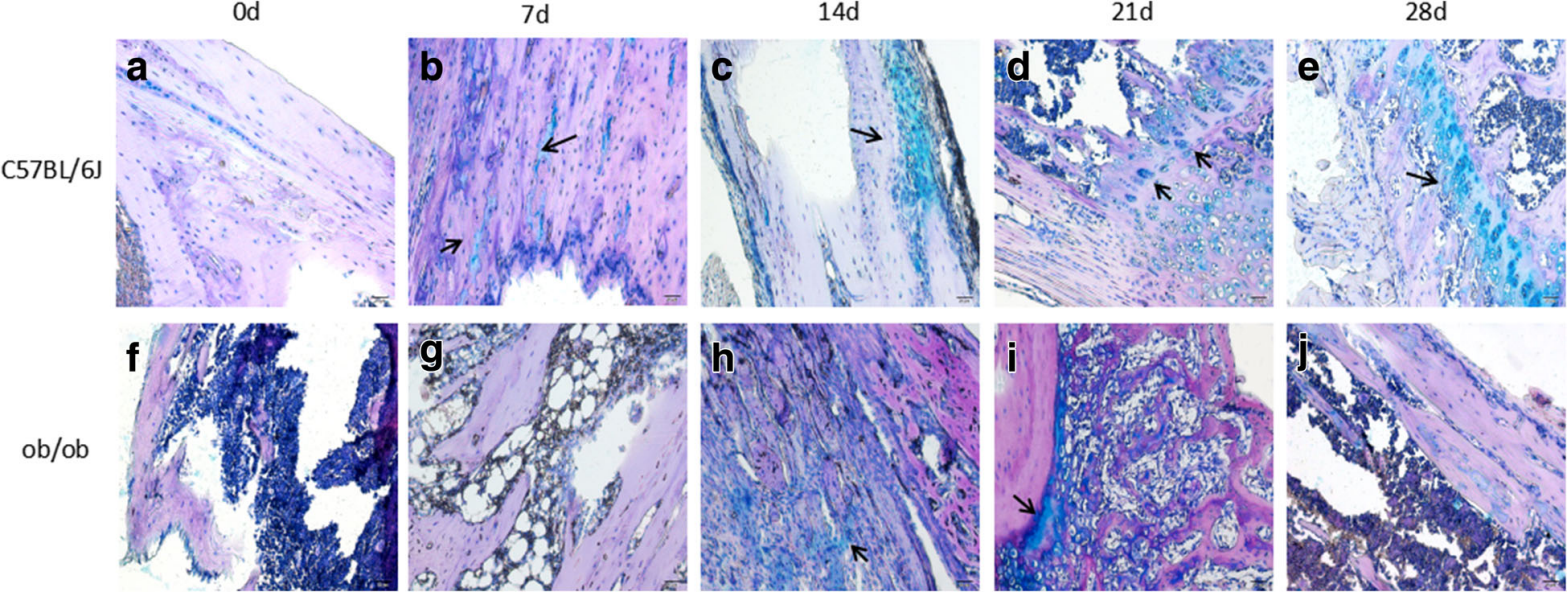
Representative optical images of AB-stained bone slides from fracture sites of normal mice (**a**, **b**, **c**, **d**, and **e**) and obese mice (**f**, **g**, **h**, **i**, and **j**) at day 0 (**a** and **f**), day 7 (**b** and **g**), day 14 (**c** and **h**), day 21 (**d** and **i**), and day 28 (**e** and **j**). Magnification × 200 and scale bar 50 μm. Black arrows point to new trabecular bones

Optical images of AB stained bone specimens were obtained at each post-fracture timepoint for each group of mice (Fig. 3). The AB staining clearly revealed that new cartilage (blue in Fig. 3b–e) was gradually formed from post-fracture day 7 for the control group of mice, indicating that bone healing was developing well. Unlike the control group, the obese mice had no new cartilage on post-fracture day 7 (Fig. 3g). New cartilage gradually formed from post-fracture day 14 (Fig. 3h), but the progress was slow (Fig. 3i, j).

### Plasma protein levels

**Fig. 4:**
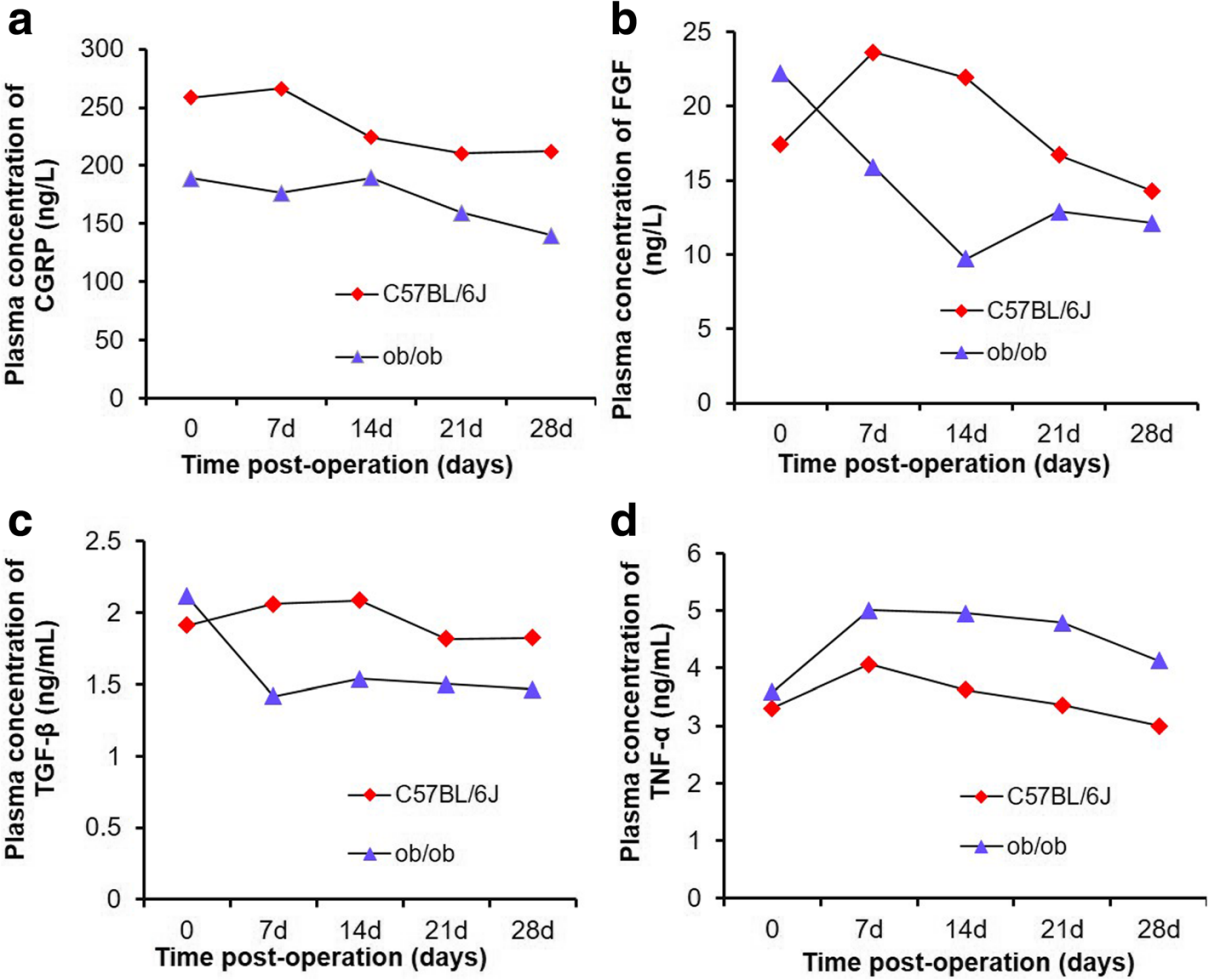
Plasma concentrations of CGRP (**a**), FGF (**b**), TGF-β (**c**), and TNF-α (**d**) in obese mice and normal mice at different timepoints post-operation

For both groups of mice, the plasma concentrations of CGRP decreased over time (Fig. 4a). However, at every postoperative timepoint, the plasma concentrations of CGRP in the control group were significantly higher than that of the obese mice.

The plasma concentrations of FGF in the control mice significantly increased from day 0 to day 7, and then consistently decreased thereafter at each timepoint (Fig. 4b). However, for the obese mice, plasma FGF levels decreased from day 0 to day 14, and then increased slightly without reaching the level at day 0. At each timepoint from days 7 to 28, the plasma concentrations of FGF in the control group were significantly higher than that of the obese mice. At day 28, the plasma concentrations of FGF in the normal control group was higher than that of the obese group.

In the control group, the plasma concentrations of TGF-β increased slightly from day 0 to day 14, and then consistently decreased at each timepoint thereafter (Fig. 4c). In the obese mice, the plasma concentrations of TGF-β decreased significantly from day 0 to day 7, and remained relatively unchanged from day 14 to day 28. At day 28, the plasma levels of TGF-β of the normal control mice were higher than that of the obese mice.

In the control group, the plasma concentrations of TGF-α slightly increased from day 0 to day 7, and then consistently decreased thereafter (Fig. 4d). In the obese mice, plasma concentrations of TNF-α rose from day 0 to day 7, gradually decreased from days 7 to day 21, and then decreased at a greater rate from days 21 to 28. At each timepoint, the plasma concentrations of TNF-α of the obese mice were higher than that of the control mice, although the differences were significant only after day 21.

## Discussion

In this study, we created tibial bone fractures in genetically obese and normal healthy mice at their right hind limb, and compared the progress in healing of both groups of mice after fixing the fractures using stainless steel intramedullary rods. In general, the fracture fixation technique used in this study results in endochondral-based bone healing [16], which consists of a series of molecular and cellular processes that are temporospatially coordinated in four stages: initial inflammatory response; soft (cartilaginous) callus formation; hard (woven bone) callus formation; and initial bony union and bone remodeling [17, 18]. Based on reports in the literature [16], we selected four timepoints to study the progress of fracture healing in the mice, specifically at 7, 14, 21, and 28 days after fracture. We set the last timepoint at 28 days, because it has been reported that at 28 to 35 days, osteoclasts populate the tissue and remodel the callus at the fracture sites of rats, converting it to a lamellar bone structure [16]. This timepoint in rats roughly corresponds to 6 to 7 weeks in humans [16]. We observed that the healing of the fractures in the obese mice was significantly slower compared with the normal mice. This was evidenced by the following observations via X-ray scans.

At 14 days post-fracture, the normal control mice had a large number of blurred shadows, indicating a large amount of callus formation (Fig. 1b), while the other group showed no obvious blurred shadows around the fracture sites, proving less formation of callus (Fig. 1f). At 21 days, the control mice had a large number of continuous blurred areas on images (Fig. 1c), and at 28 days, continuous cortical bone at the fracture site (Fig. 1d). However, at both 21 and 28 days, the obese mice showed less volume of callus and had no continuous cortical bones at the fracture sites (Fig. 1g, h). This delayed healing of fractures in obese mice was consistent with previous reports [19].

The results from the H&E staining experiments further confirmed the slow or delayed healing of bone fractures in the obese mice. The formation of stabilizing callus is the key step for fracture healing, in which cartilage is formed, then resorbed, and finally replaced with new bone [20]. In the present study, at 21 days after fracture, the control mice already showed visible trabecular bone at the fracture site (Fig. 2d), while for obese mice, visible trabecular bone was absent (Fig. 2i). At post-fracture day 28, the fracture ends in the control mice were connected by bone scabs which were filled with well-arranged trabecular bone and osteoblasts (Fig. 2e). For obese mice, there was less cartilage callus, and bone callus had increased at the fracture sites (Fig. 2g). These observations are in accord with reports from other research groups [21].

It has been reported that, for bone healing, it is essential that mesenchymal cells and chondro-osteoprogenitor populations are recruited to the fracture site [17, 22, 23]. In the present study, during the initial 7 days after fracture, the normal mice apparently had more chondrocytes and undifferentiated mesenchymal cells than did the obese mice. At 28 days, osteoblasts had filled the bone scabs (Fig. 2e) in this group.

The slow or delayed healing of bone fractures in the obese mice was also confirmed by the AB staining, which showed that the time to form new cartilage in obese mice was 7 days later than for normal mice, and new cartilage formation progressed much more slowly than for normal mice.

The recruitment of mesenchymal cells and their subsequent differentiation into osteoblasts are significantly important for bone healing. However, these two steps require growth factors for angiogenesis, neovascularization, and promoting new bone formation. In the present study, we therefore quantified the concentrations of CGRP, FGF, and TGF-β1 in the mouse blood. These proteins are well recognized for their roles in bone healing. At each timepoint post-fracture, the normal mice had a higher plasma concentration of CGRP than did the obese mice. High levels of CGRP in serum released from brain tissue after traumatic brain injury has been shown to enhance fracture healing [24]. Another recent study showed that local neuronal production of CGRP induced by magnesium implants improved the healing of bone fracture in rats [25]. Therefore, our observations of both low plasma CGRP concentration and delayed fracture healing in obese mice is consistent with these earlier reports.

It has been reported that FGF-2 chemically controlled released from tissue engineering constructs significantly increased bone formation in a mice critical-sized calvarial defect model [26]. In addition, overexpression of FGF-2 in transgenic mice also accelerated bone formation via faster progression through the stages of cartilage formation, bone union, and callus remodeling with higher numbers of osteoblasts around the fracture area [27].

These previous reports confirm that the lower plasma FGF concentration in the obese mice of the present study, observed at all timepoints, strongly contributed to the slower fracture healing in the obese mice compared with the normal group. Indeed, the plasma FGF concentration in the control group increased from baseline after the surgery until at least day 14. This short-term increase in plasma FGF concentration benefited fracture healing in the normal mice.

For TGF-β1, the changes over time in its plasma concentration in the control group were similar to that reported for normal healing in humans, i.e., initially increasing and then decreasing thereafter [28]. However, in the obese group of the present study, plasma TGF-β1 levels decreased during the initial period, with a subsequent slight increase at 14 days, and then continuously decreased. The plasma TGF-β1 level in the obese mice never reached the corresponding level in the normal mice at any timepoint—7, 14, 21, or 28 days post-surgery. An earlier study reported that fracture healing in adult male rats could be accelerated by increased expression of TGF-β1 in both plasma and at the fracture site, when due to traumatic brain injury [29]. Another study revealed that the administration of naproxen sodium into bone fracture model rats decreased their TGF-β1 serum levels and resulted in a slow fracture healing for these rats, while the use of granulocyte colony stimulating factor (G-CSF) increased the TGF-β1 serum levels and led to a better fracture healing [30]. In addition, TGF-β1 released from TGF-β1-loaded microgranules promoted bone regeneration in rabbit calvarial defects after 4 weeks [31]. Therefore, it is reasonable to believe that, in the present study, the lower plasma concentration of TGF-β1 in the obese mice observed at all timepoints (7, 14, 21, and 28 days post-surgery) was a contributing factor to the slower fracture healing in the obese mice compared with the normal group.

Regarding the mechanism of slower healing in the obese mice of the present study, the lower plasma concentrations of TGF-β1 and FGF in the obese group were associated with slower formation of blood vessels at the fracture sites. This in turn contributed to less recruitment of mesenchymal cells and chondrocytes, also observed in these mice. Moreover, lower plasma concentrations of CGRP, FGF, and TGF-β1 apparently inhibited further the proliferation of mesenchymal cells and differentiation of mesenchymal cells into bone-forming cells such as osteoblasts, as few or no osteoblasts were observed in the obese group at 28 days. These results are consistent with earlier reports concerning the functions of CGRP [25, 32], FGF [27, 33], and TGF-β1 [28, 34, 35].

In this study, the obese group showed higher levels of plasma TNF-α compared with the normal control mice at all timepoints. This higher level of TNF-α in the obese group is consistent with earlier reports that TNF-α is highly expressed in obese children [36–39], and increased levels of TNF-α were detected in the serum of obese mice induced by a high-fat diet [40]. In addition, bone fractures immediately initiate inflammatory responses, thus further stimulating the secretion of various inflammatory factors, including TNF-α. In the present study, plasma TNF-α levels increased in both groups during the initial 7 days post-fracture, but the obese mice maintained their higher plasma TNF-α levels longer than did the normal control mice. This longer duration may have promoted the formation and differentiation of osteoclasts from mesenchymal cells [41, 42]. This might further have contributed to the delayed bone healing in the obese group. Thus, the delayed bone healing in the obese group is in accordance with the observation from other research groups that a TNF-α blockade improved tendon-bone healing in rats at early timepoints [43].

This study is limited by its relatively small subgroup size and few observed timepoints.

## Conclusions

Our results suggest that bone fracture healing was significantly slower in the obese mice relative to that of the normal mice. The lower levels of CGRP, FGF, and TGF-β, and higher levels of TNF-α, in the obese mice may have contributed to this delay in fracture healing.

## Abbreviations

AB: Alcian blue
CGRP: Calcitonin gene-related peptide
ELISA: Enzyme-linked immunosorbent assay
FGF: Fibroblast growth factor
H&E: Hematoxylin and eosin stain
TGF-β1: Growth factor beta 1
TNF-α: Tumor necrosis factor-α
